# Advancing lasers in silicon photonics

**DOI:** 10.1038/s41377-023-01252-w

**Published:** 2023-09-04

**Authors:** Daoxin Dai

**Affiliations:** State Key Laboratory for Extreme Photonics and Instrumentation, College of Optical Science and Engineering, International Research Center for Advanced Photonics, Zhejiang University, Zijingang Campus, Hangzhou, 310058 China

**Keywords:** Lasers, LEDs and light sources, Optical physics

*Nature* **620**, 78–85 (2023)

10.1038/s41586-023-06251-w

The integration of lasers has long been a significant challenge, impeding advancements in silicon photonic integrated circuits (PICs). Although the majority of applications necessitate lasers, most silicon PICs still need external off-chip lasers, as on-chip lasers have yet to match the performance of discrete counterparts. Additionally, stable laser operation requires adequate isolation from downstream reflections, which can negatively influence the performance; thus, isolators are typically introduced between a laser and a silicon PIC. Now, a group of researchers from the University of California, Santa Barbara, along with collaborators from other institutions, have developed a novel approach to address this issue: three-dimensional (3D) photonic integration. This technique enables the integration of high-performance lasers and ultra-low-loss waveguides on a silicon PIC, yielding Hertz-linewidth lasers with high resistance to downstream reflections. These lasers have the great potential to advance silicon PICs in numerous applications, particularly those with stringent noise requirements. Furthermore, the 3D structure integrating active and passive components on silicon may unlock the full potential of photonic integration, paving the way for highly robust photonic chips with comprehensive functionality and integrity.

